# Low prevalence of irritable bowel syndrome in primary health care in four Swedish counties

**DOI:** 10.3109/02813432.2013.811949

**Published:** 2013-09

**Authors:** Rasmus Waehrens, Henrik Ohlsson, Jan Sundquist, Kristina Sundquist, Bengt Zöller

**Affiliations:** ^1^Center for Primary Health Care Research, Lund University, Malmö, Sweden; ^2^Stanford Prevention Research Center, Stanford University School of Medicine, Palo Alto, California, USA

**Keywords:** Epidemiology, gender, general practice, irritable bowel syndrome, prevalence, primary health care, Sweden

## Abstract

**Objective:**

Few large-scale studies have examined the prevalence of irritable bowel syndrome (IBS) and the number of visits among IBS patients in a primary health care setting. The aim of this study was to assess the prevalence of IBS in primary health care in four Swedish counties. Another aim was to study the number of visits among the IBS patients.

**Design:**

A register-based study.

**Setting:**

A primary health care database with information on patients from 71 primary health care centres in the Swedish counties of Stockholm, Uppsala, Värmland, and Gotland.

**Subjects:**

The primary health care database contains individual-level data for 919 954 patients for the period 2001–2007.

**Main outcome measures:**

Prevalence of IBS diagnosis.

**Results:**

10 987 patients had a diagnosis of IBS, which corresponds to a prevalence of 1.2%. IBS was most common in the 25–44 years age group (37% of IBS patients); 71% of IBS patients were female, and 81% of IBS patients visited their GP six or more times, compared with 46% of non-IBS patients. However, 95% of the IBS patients visited their GP three times or fewer for IBS.

**Conclusion and implications:**

The prevalence of IBS was low among Swedish primary health care patients. This might suggest that IBS patients are insufficiently diagnosed in Swedish primary health care.

- Few large-scale studies have examined the prevalence of irritable bowel syndrome (IBS) among unselected patients in primary health care.
- The prevalence of IBS diagnoses in Swedish primary health care was low (1.2%).
- IBS patients often visited their GP, but rarely because of IBS.

## Introduction

Irritable bowel syndrome (IBS) is a functional gastrointestinal (GI) disease characterized by chronically recurring abdominal pain or discomfort and altered bowel habits [1,2]. It has been reported to be one of the most common gastrointestinal disorders, with a worldwide prevalence of 2.5% to 25% [1,2]. The pathogenesis of IBS remains incompletely understood [1,2]. The pathophysiology is probably multifactorial, with involvement of both genetic and environmental factors. Suggested mechanisms include psychosocial factors, abnormal gastrointestinal motility, visceral hypersensitivity, mucosal inflammation after gastroenteritis, and small intestinal bacterial overgrowth [1,2]. Four different sets of diagnostic criteria for IBS have been used: the Manning criteria, the Rome I criteria, the Rome II criteria, and the Rome III criteria [1,2]. The existence of these different criteria poses problems for the comparison of prevalence studies over time. Moreover, in primary health care a more pragmatic approach to diagnosis, involving clinical judgement rather than specific criteria, is usually adopted [3].

Population-based surveys from Europe and the US have shown the prevalence of IBS to be 7–12.5% [4–8]. The prevalence was higher among females than males: the gender ratio was about 2:1 [4–8]. In another population-based survey, the prevalence of IBS varied from 5.1% to 16.2% depending on whether the diagnosis of IBS was based on the Manning or Rome I or II criteria [9]. In a community survey in the US, the overall prevalence of IBS was 14.1% [10]. Of the IBS patients identified in that study, only 23% had previously been medically diagnosed [10]. Among 3111 patients seen by 36 general practitioners (GPs) at six locations in and around Bristol, UK, only 2.5% were judged to have IBS [11]. This is a much lower prevalence than those obtained in most population-based studies [4–10]. Rather, it is more similar to the figure of 1.6% obtained in an older nationwide study based on data from six systematic national health surveys and registers in the US [12].

The discrepancy in IBS prevalence between population-based studies and primary health care-based studies [4–12] may not only be due to diagnostic differences [3]. It might also be related to health-care-seeking behaviours of IBS patients, as reviewed by Spiller et al. [2]. In many studies, only around 50% of IBS patients are diagnosed [2]. The main predictors of health-care seeking among IBS patients are abdominal pain or distension, pain severity, symptoms according to the Rome II criteria, and psychological and social factors [2]. IBS patients tend to seek health care more often than non-IBS patients [2]. IBS has been reported to be a risk factor for becoming a frequent health care attender [13]. Frequent health care attenders often have psychosocial problems [13,14]. In line with this, IBS has been associated with comorbidities such as depression, anxiety, fibromyalgia, headache, migraine, and lower urinary tract symptoms (LUTS) [2,15,16].

There have been few recent large-scale primary health care register studies of IBS. This study was conducted to examine the prevalence of IBS and number of visits among IBS patients using a large primary health care database. Our hypothesis was that there would be age and gender effects on the prevalence of IBS and that certain comorbidities would be associated with IBS.

## Material and methods

This study was approved by the Ethics Committee of the Karolinska Institute, Huddinge, Sweden (reference number 12/2000, 2000-03-06 and 2002-11-18) and was performed in compliance with the Helsinki Declaration. The study population was from a primary health care database covering 71 primary health care centres in the Swedish counties of Stockholm (n = 687 310), Värmland (n = 145 943), Gotland (n = 84 898), and Uppsala (n = 12 790). The primary health care database contains individual-level data from a total of 919 954 individuals who visited their GP during the period 2001–2007.

Cases of IBS diagnosed by GPs were identified by the International Classification of Diseases (ICD-10) code K58. Five comorbidities known to be associated with IBS were selected in order to evaluate whether the patients with IBS diagnoses in the present study had the same comorbidity patterns as those described in previous literature [2,15,16]. These comorbidities were defined by the following ICD-10 codes: depression (F32, F33, and F412); LUTS (R30); migraine (G43); headache (R519 and G442); and fibromyalgia (M797). However, fibromyalgia was not included in the analyses as no IBS patients in the database were also diagnosed with fibromyalgia. Age, gender, and number of GP visits were also included in the analysis.

### Statistical analysis

Logistic regression was used to investigate the associations between IBS and gender, age, number of GP visits, and comorbidities. Odds ratios (ORs) and corresponding 95% confidence intervals were calculated. Three main models were used in the logistic regression analysis of the data in the primary health care database, with IBS as the outcome. In model A, only age and gender were included and their associations with IBS were analysed. In the B models (B1–B5), associations between number of GP visits and different comorbidities among the IBS patients were analysed. Gender and age were controlled for in all B models. In model B1, which was controlled for age, the association between number of GP visits and IBS was studied. Models B2, B3, B4, and B5 analysed the associations of IBS with depression, LUTS, migraine, and headache (including an interaction term with gender), respectively (with all models being controlled for gender and age). In model C, gender, age, number of GP visits, and comorbidities were included. All calculations were performed using SAS version 9.2.

## Results

### Primary health care database

Table I shows descriptive statistics for all 919 954 individuals included in the primary health care database, which contains information on all GP visits between 2001 and 2007. The age and gender distribution (47% male), number of GP visits, and four comorbidities known to be associated with IBS (depression, migraine, LUTS, and headache) are shown. Individuals aged 0–24 years constituted the largest age group, accounting for 35% of all patients. Depression was diagnosed in 5% of all patients, and 47% of all patients visited their GP six times or more.

**Table I.** Descriptive statistics for all 919 954 individuals in the primary health care database.

	All patients	Patients without IBS	Patients with IBS
Number of patients	919 954 (100)	908 967 (98.8)	10 987 (1.2)
Age (years):			
0–24	323 221 (35)	320 984 (35)	2 237 (20)
25–44	271 991 (30)	267 948 (30)	4 043 (37)
45–64	210 108 (23)	206 874 (23)	3 234 (29)
65–74	62 506 (7)	61 563 (7)	943 (9)
75–84	40 344 (4)	39 892 (4)	452 (4)
85+	11 110 (1)	11 038 (1)	72 (1)
Male	430 759 (47)	427 560 (47)	3 199 (29)
Number of GP visits:			
1–2	270 724 (29)	270 020 (30)	704 (6)
3–5	217 744 (24)	216 373 (24)	1 371 (12)
6+	431 486 (47)	422 574 (46)	8 912 (81)
Depression (F32, F33, F412)	44 992 (5)	43 345 (5)	1 647 (15)
Lower urinary tract symptoms (R30)	3 257 (0.4)	3 154 (0.4)	103 (1)
Migraine (G43)	12 047 (1)	11 659 (1)	388 (4)
Headache (R519, G442)	8 699 (1)	8 315 (1)	384 (4)

Note: Data are presented as n (%).

### IBS in the primary health care database

**Figure 1.**
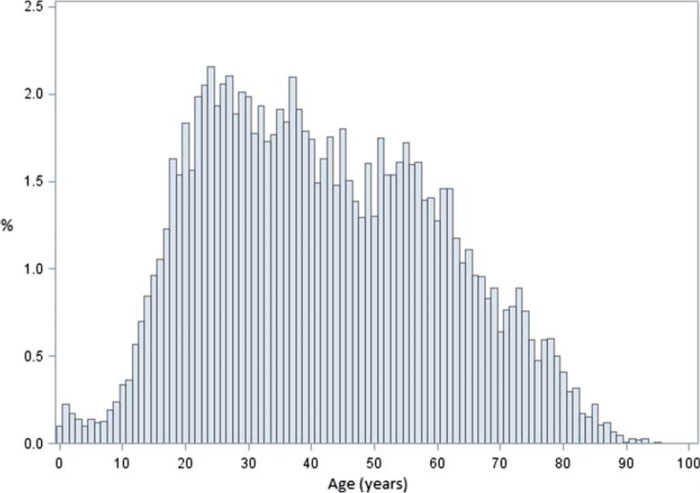
Age distribution of individuals with IBS (n = 10 987).

Of the 919 954 patients, 10 987 (overall prevalence 1.2%) had a diagnosis of IBS (see Table I). Of the IBS patients, 29% (n = 3 199) were male (see Table I). The age distribution for IBS patients is shown in Table I and Figure 1. The mean age of IBS patients at first diagnosis was 41.9 years (SD 18.7 years; range 0–95 years) and 57% of IBS patients were younger than 45 years. IBS patients visited their GP frequently: 81% of IBS patients visited their GP six or more times between 2001 and 2007, compared with 47% of non-IBS patients. Some 15% of IBS patients had been diagnosed with depression by their GP (see Table I).

### Yearly prevalence and incidence from 2001 to 2007

The 12-month prevalence did not vary greatly during the study period (2001–2007). The highest 12-month prevalence was 0.55% in 2004 and the lowest was 0.44% in 2001. The estimated yearly incidence, defined as first registration during the study period, also varied little during the study period. The highest yearly incidence was 4.4 per person-year in 2004 and the lowest was 3.7 per person-year in 2007.

### Prevalence of IBS in the four different counties included in the study

The seven-year prevalences of IBS in the four different counties were 1.2% (95% CI 1.2–1.2) for Stockholm, 1.1% (95% CI 1.1–1.2) for Värmland, 1.0% (95% CI 0.9–1.1) for Gotland, and 2.9% (95% CI 2.5–3.1) for Uppsala.

### Logistic regression analysis of factors associated with IBS

Table II shows the results of the logistic regression analysis. Three models were used. In model A, only age and gender were included. Male gender was associated with decreased odds of IBS. Individuals aged 25–44, 45–64, and 65–74 years had the highest ORs compared with the reference group (Table II).

**Table II.** Results from logistic regression analysis of odds of IBS using data for the 919 954 individuals in the primary health care database.

	Model A	Models B1–B5	Model C
Gender (male vs. female)	0.47 (0.45–0.49)	–	0.54 (0.52–0.58)
Age (years):			
0–24	1 (Ref)	–	1 (Ref)
25–44	2.12 (2.02–2.24)	–	1.85 (1.75–1.95)
45–64	2.23 (2.11–2.35)	–	1.61 (1.52–1.70)
65–74	2.16 (2.00–2.33)	–	1.39 (1.28–1.50)
75–84	1.52 (1.38–1.69)	–	0.93 (0.84–1.03)
85+	0.82 (0.65–1.04)	–	0.56 (0.44–0.70)
Number of GP visits:			
1–2	–	1 (Ref)	1 (Ref)
3–5	–	2.41 (2.20–2.64)	2.36 (2.16–2.59)
6+	–	7.65 (7.08–8.26)	6.91 (6.39–7.47)
Depression (F32, F33, F412)	–	2.76 (2.61–2.91)	1.81 (1.71–1.91)
Lower urinary tract symptoms (R30)	–	2.54 (2.08–3.09)	1.79 (1.47–2.19)
Migraine (G43)	–	2.15 (1.94–2.39)	1.34 (1.21–1.49)
Headache (R519, G442) (males)	–	4.50 (3.58–5.65)	2.76 (2.19–3.47)
Headache (R519, G442) (females)	–	2.95 (2.63–3.33)	1.79 (1.59–2.02)

Notes: Data are presented as OR (95% CI) for diagnosis of IBS. In model A, only age and gender were included. In the B models (B1–B5), associations of IBS with number of GP visits and different comorbidities were analysed. Gender and age were controlled for in all B models. In model B1, the association between number of GP visits and IBS was studied. Models B2–B5 analysed the associations of IBS with different comorbidities. In model C, gender, age, number of GP visits, and comorbidities were included.

In models B1–B5 (controlled for age and gender), all comorbidities were associated with IBS, with headache among males having the highest OR – higher than the OR for headache among females. No other significant gender differences were identified (data not shown). The OR for IBS was high among those who made six or more GP visits.

In model C, gender, age, number of GP visits, and comorbidities were included. The observed associations were similar to those obtained using models A and B. Six or more GP visits was the factor with the strongest association with IBS in model C.

### Number of visits for IBS among IBS patients

In total 95% (10 462) of the IBS patients made between one and three GP visits for IBS. Only 5% (525) of the IBS patients made four or more visits for IBS. Males with IBS tended to have lower odds than females of four or more GP visits, but the difference was not significant (data not shown). In a multivariate model, only increasing age and depression were significantly associated with four or more GP visits for IBS (data not shown).

## Discussion

### Statement of principal findings

This is the first Swedish study to assess the prevalence of IBS using data from a large primary health care register. We found the prevalence of IBS to be only 1.2%, much lower than in many previously published studies [4–10]. However, our value is in line with a study that did not find IBS to be a common minor ailment in out-of-hours primary care [17]. Ninety-five percent of IBS patients visited their GP three times or fewer during the study period. Similar to previous studies, the IBS patients in the present study visited primary health care more often for non-IBS problems than for IBS [2]. Moreover, IBS patients made more GP visits for other conditions than patients without IBS. IBS was associated with depression, migraine, LUTS, and headache, in accordance with previous studies [2,15,16] and in line with the notion that psychological factors may be involved in the pathogenesis of IBS [1,2]. Surprisingly, IBS was not associated with fibromyalgia, which was previously described in IBS patients in primary care [18]. As in other studies, the majority of IBS patients were young females [1,2].

### Strengths and weaknesses of the study

One strength of this study is the use of a large primary health care database containing information on all primary health care visits in well-defined areas. This approach eliminated any selection bias. The study is, however, limited by the fact that the diagnostic criteria used are unknown. A pragmatic approach to diagnosis, involving clinical judgement rather than specific criteria, is usually adopted in primary care [3]. IBS is used by many GPs as a diagnosis after exclusion of other conditions [19]. Also, the diagnosis of IBS has not been validated in our database. A general-practice-based database in the UK has been extensively validated. The positive predictive value of an IBS diagnosis in the UK database was 77% [20]. The gender and age distribution and associated comorbidities are similar to those in other studies of IBS [1–12]. This may indirectly suggest that the ICD-10 code K58 mostly identifies IBS patients in the primary health care database. The fairly similar prevalences of IBS in the four different counties represented in the database are also reassuring of relatively good diagnostic validity.

### Strengths and weaknesses in relation to other studies

Few large-scale studies have determined the prevalence of IBS in primary health care [11], which was the aim of the present study. Many studies estimating the prevalence of IBS are population-based studies with defined diagnostic criteria [4–10], which do not reflect a primary health care setting [11]. Strengths of these studies are, however, the use of predefined criteria such as the Manning or Rome criteria [1,2,4–10]. However, these criteria have some limitations as they have different sensitivities for IBS diagnosis. The IBS prevalence of 1.2% in the present study is more similar to the prevalence of 2.5% from a previous primary health care study [11] than those from population-based surveys [4–10]. Our results show that IBS patients visit their GP more often than non-IBS patients, which further supports the idea that people with IBS may use more health care resources than people without IBS [2,13]. As described in the literature, a significant number of children with IBS were also identified [21].

### Meaning of the study

The low prevalence of IBS in this study may be due to GPs not being familiar with the Manning or Rome I, II, or III criteria [2,3,19]. Moreover, it has been shown that functional disorders are underreported in Swedish primary health care [22]. It has been suggested that GPs may have insufficient knowledge in diagnosing and handling functional disorders such as IBS [23]. GPs may also be reluctant to use stigmatizing diagnoses [23], and they may also find that there is insufficient time to manage patients with functional disorders [23]. This hypothesis is further underlined by the lack of an association between IBS and fibromyalgia in the present study, which contradicts a previous report [18].

The associations with comorbidities and an increased number of non-IBS GP visits are in line with previous research showing that people with IBS have lower health-related quality of life (HRQOL) scores [24] than those without IBS. This suggests that IBS patients may be insufficiently diagnosed and inadequately treated in primary care in Sweden. This explanation is more likely than the alternative one: that the actual prevalence of IBS is low in Swedish primary health care.

In conclusion, the prevalence of IBS diagnoses was low in this study from Swedish primary health care. IBS patients visited their GP often, but rarely because of IBS.

### Unanswered questions and future research

This study suggests clinically relevant topics for research on IBS in primary health care, and raises the question as to why the prevalence of IBS diagnoses is so low in primary health care. As well as answering this question, future studies may also highlight the role of patient questionnaires in the diagnosis of IBS in primary health care.
